# Leveraging Artificial Intelligence to Appraise Social Media Narratives on Breast Reduction Procedures: A Snapshot Analysis

**DOI:** 10.7759/cureus.117785

**Published:** 2026-10-11

**Authors:** Maria P Farez Ochoa, Henry K Mah, Mayesha Ahmed, Mariam Zafar Khan, Gabriel De la Cruz Ku, Joseph M Escandon, Andrew Peredo

**Affiliations:** 1 Surgery, Saba University School of Medicine, The Bottom, BES; 2 Internal Medicine, Wyckoff Heights Medical Center, New York City, USA; 3 Plastic and Reconstructive Surgery, Universidad Cientifica del Sur, Lima, PER; 4 General Surgery, Wyckoff Heights Medical Center, New York City, USA; 5 Plastic and Reconstructive Surgery, Huntington Hospital, Huntington, USA

**Keywords:** breast surgery, communication, mammaplasty, plastic and reconstructive surgery, social media

## Abstract

Background: Social media platforms increasingly influence patient perceptions regarding reconstructive procedures. This study evaluated the characteristics, quality, engagement patterns, and user sentiment associated with breast reduction-related content on TikTok, while assessing the performance of artificial intelligence (AI) in content and sentiment analysis.

Methods: A cross-sectional analysis of 140 TikTok videos on breast reduction was conducted. Engagement metrics, including likes, comments, shares, saves, and a weighted engagement index, were collected. The modified DISCERN (mDISCERN) and Global Quality Scale (GQS) were used to appraise content quality by AI and two human reviewers. Sentiment analysis was performed by human reviewers, ChatGPT, and Claude AI. High-engagement videos were defined as those within the upper quartile for likes (≥30,400) or shares (≥1,050).

Results: Most videos were created by patients (89%) and focused on postoperative experiences (75%). Videos demonstrated substantial engagement, with median values of 3,860.5 likes, 131 comments, and 194.5 shares. Overall informational quality was poor, with median GQS and mDISCERN scores of 1-2 across raters. Highly liked and highly shared videos were significantly shorter in duration (18 vs. 34 seconds; p < 0.05) and generated markedly greater engagement across all metrics. Popular videos received significantly lower quality scores from human reviewers. Sentiment analysis revealed predominantly positive responses (49-58%). Claude showed greater concordance with human consensus than ChatGPT (κ = 0.749 vs. 0.649).

Conclusion: Breast reduction content on TikTok is largely patient-driven and characterized by high engagement but low informational quality. Shorter videos achieved greater dissemination despite being associated with less reliable health information.

## Introduction

Social media platforms, particularly TikTok, Instagram, and Facebook, have transformed how patients seek and consume information about plastic surgery [1]. While these platforms have democratized access to surgical information, they have simultaneously propagated unrealistic beauty standards through filtered imagery and trend-driven content, fostering misconceptions about risks, limitations, and individualized candidacy [2,3]. Patients increasingly request procedures based on viral trends rather than clinical appropriateness, underscoring a growing gap between social media portrayals and evidence-based medicine [2,3].

Breast reduction surgery remains among the most consistently performed and patient-satisfying procedures in plastic surgery [4,5]. According to the American Society of Plastic Surgeons, 76,734 reduction mammaplasty procedures were performed by member surgeons in 2024, reflecting year-over-year growth and an evolving patient landscape [6]. As procedural volumes rise, understanding the factors that shape patient expectations and decision-making becomes increasingly important [6,7].

Artificial intelligence (AI) has emerged as a powerful tool capable of systematically analyzing large volumes of data, including social media content, with applications extending to scientific inquiry [8]. As plastic surgeons, harnessing AI's analytical capabilities while critically evaluating its limitations is essential for advancing patient education and specialty integrity [8]. Therefore, this study utilized two different AI tools to systematically evaluate breast reduction-related content on TikTok, one of the fastest-growing social media platforms shaping public perceptions of surgical procedures. The primary objectives were to assess the informational quality of reduction mammaplasty-related videos and characterize prevalent content trends. Our secondary objectives were to identify which post characteristics were associated with user engagement and to analyze the sentiment of user responses to this content.

## Materials and methods

This observational, cross-sectional study set out with the central aim of methodically mapping the nature of reduction mammaplasty-related material circulating on the TikTok platform (ByteDance Ltd., Beijing, China), emphasizing content categories, characteristics of the individuals posting, and the engagement levels achieved by the most visible videos [9]. As a secondary aim, the study sought to measure specific forms of user interaction, including likes, shares, and the tone of comments, in order to shed light on how the public generally perceives reduction mammaplasty within this particular social media environment [2,3]. TikTok makes video-level engagement metrics publicly visible, which allows a standardized engagement analysis. Inclusion was restricted to videos either presented in English or featuring English-language voiceover or narration. On May 5-6, 2026, we conducted a search in the New York City area using the search tool in the TikTok mobile application. We searched for the term "BreastReduction" and selected the top 140 videos from the "Top" tab. This study used only publicly available data and was conducted in accordance with the ethical principles of the Declaration of Helsinki [2,3]. Ethical exemption for this study was granted by the Institutional Review Board (#WKF315123-21), given that it did not meet the criteria for human subjects research.

The top 140 videos tagged with #BreastReduction were located, and each was systematically coded for a range of variables, including engagement metrics (shares, likes, comment volume), co-occurring hashtags, time elapsed since posting, defining features of the video content itself, and the category of creator responsible for the post [2,3]. Given that likes, comments, and shares each capture a functionally different type of engagement, with comments and shares arguably signaling deeper cognitive investment, more active participation, and broader dissemination of information relative to the comparatively passive act of liking, a Weighted Engagement Composite Index was constructed to yield a more holistic measure of audience interaction [3,10,11]. This index was calculated using the following formula [10,11]:

$\text{Engagement Index} = \frac{(\mathrm{Likes}\times1) + (\mathrm{Comments}\times3) + (\mathrm{Shares}\times2)}{\text{Video Creator Followers}}$

Creators were sorted into four distinct, nonoverlapping groups: (i) surgeons holding board certification, (ii) individuals who had undergone the procedure (patients), (iii) healthcare professionals who were not physicians, and (iv) advocates or members of the general public not belonging to any of the preceding categories [2,3].

A structured, supervised coding scheme was used to classify content according to predetermined thematic categories: educational (material conveying factual information about reduction mammaplasty), preoperative experience (content covering considerations or expectations in the lead-up to surgery), postoperative experience (material illustrating recovery, aftercare, or surgical outcomes), and awareness/support/humor (content aimed at promoting, destigmatizing, or humorously portraying reduction mammaplasty or breast reduction more broadly). To safeguard the reliability of this classification, a two-reviewer verification process was paired with an artificial intelligence-supported categorization system [2,3].

Videos with a minimum of 30,400 likes or at least 1,050 shares were designated as high-engagement content, figures that represented the top quartile (75th percentile) for both metrics across the full sample of videos evaluated in this study.

Quality evaluation measures

Two previously validated instruments were used to assess the quality of information conveyed in each video: the modified DISCERN tool (mDISCERN) and the Global Quality Scale (GQS) [12,13]. The GQS, structured as a five-point Likert scale, gauges online health content in terms of its overall clarity, thoroughness, and value as an educational resource (Appendix 1). The mDISCERN instrument, derived from the original DISCERN framework, is designed specifically to probe indicators of trustworthiness in digital health communication, such as whether sources are properly attributed, whether risks and benefits are disclosed, whether the information presented is balanced, and whether references are provided (Appendix 2). Scores for both the GQS and mDISCERN were generated through a combined approach that paired human raters with an artificial intelligence system powered by a large language model (LLM), specifically ChatGPT Free running GPT-5.5 Instant (OpenAI, San Francisco, California). To facilitate AI-assisted scoring, transcripts of each video were first produced using Evernote's AI Transcribe tool (Evernote Corporation, Redwood City, California), after which these transcripts were used as input for the quality assessment process. Prior to analysis, all transcripts were reviewed manually to identify and account for any significant transcription inaccuracies.

Sentiment analysis of extracted comments

For each video, the top 10 comments left by users were extracted in order to evaluate their sentiment. Two human reviewers (M.A. and M.Z.K.) independently determined the polarity of this sentiment, categorizing it as positive, negative, or neutral, using a structured framework designed for sentiment analysis (Appendix 3) [14]. To standardize sampling given the considerable variability in comment volume across videos, a set number of comments was chosen for each. This strategy allowed for consistent representation of every video while preventing videos with unusually high comment counts from being disproportionately represented. Agreement between the two raters was assessed via Cohen's kappa coefficient (κ), interpreted along a scale ranging from ≤0 (indicating no agreement) to 1.0 (indicating perfect agreement) [15]. Only those comments on which both human reviewers were in full agreement were carried forward for comparative analysis using artificial intelligence.

AI-powered sentiment analysis

Following the manual review process, sentiment classification of the comments was performed with the aid of two large language models, ChatGPT (OpenAI, San Francisco, California) and Claude Sonnet 4.6 (Anthropic PBC, San Francisco, California). Both systems, having undergone pretraining on extensive linguistic datasets and subsequent fine-tuning for context-aware sentiment detection, applied a context-sensitive, rule-based approach to categorize each comment as expressing positive, negative, or neutral sentiment [16]. Prior to analysis, the models were provided with a domain-specific vocabulary, relevant contextual indicators, and preset thresholds for classification (Appendix 3), which allowed for more refined detection of subtle sentiment cues, such as emojis, figurative language, and other paralinguistic signals.

Statistical analysis

To ensure full reproducibility under the defined analytical conditions, the AI-driven analysis was conducted independently on two separate occasions, each using a different access date, user account, and network environment; only outputs that matched exactly between the two runs were retained for the final dataset. Relationships among content type, creator category, and engagement metrics (likes, shares, comments, hashtags) were evaluated through descriptive and inferential statistical methods. Because the data were nonparametrically distributed, the Mann-Whitney U test was applied to compare medians between groups. Agreement between raters was quantified using Cohen's κ, reported with 95% confidence intervals (95% CI); values falling within the 0.81-1.0 range were considered indicative of near-perfect agreement. A p-value below 0.05 was set as the threshold for statistical significance.

## Results

A total of 140 TikTok videos related to breast reduction were identified and included in the analysis. The overall characteristics of the included videos are summarized in Table 1. The majority of the content was created by patients (124; 89%) and surgeons (11; 8%). In terms of content type, postoperative experiences constituted the predominant category (105; 75%), followed by educational content (14; 10%), preoperative experiences (12; 9%), awareness/humor/support videos (6; 4%), and procedural content (3; 2%). Videos had a median duration of 28.5 seconds (IQR: 14-60.25) and had been posted a median of 171 days prior to data collection (IQR: 60-546.75). 

**Table 1:** Overview of the characteristic of included videos. IQR: interquartile range; GQS: Global Quality Scale; AI: artificial intelligence; mDISCERN: modified DISCERN.

Variables	Frequency (percentage)/median (IQR)
Type of author	
Patient	124 (89%)
Surgeon	11 (8%)
Non-MD healthcare	3 (2%)
Activist	2 (1%)
Type of content	
Postoperative experience	105 (75%)
Education	14 (10%)
Preoperative experience	12 (9%)
Awareness/humor/support	6 (4%)
Procedure	3 (2%)
Duration of post (sec)	28.5 (14-60.25)
Age of video (days)	171 (60-546.75)
Likes	3860.5 (863-30,475)
Number of comments	131 (35-318.5)
Users saving post	400 (90.75-1654.5)
Hash-tags	3 (2-5)
Shares	194.5 (37.75-1046.75)
Share/day index	1.33 (0.29-4.91)
Followers	11400 (2241-45,400)
Engagement index	0.44 (0.06-5.46)
GQS scores	
GQS Human #1	1 (1-2)
GQS Human #2	2 (1-2)
GQS AI	2 (1-2)
mDISCERN scores	
mDISCERN Human #1	1 (1-2)
mDISCERN Human #2	1 (1-2)
mDISCERN AI	1 (1-2)

The median number of likes per video was 3,860.5 (IQR: 863-30,475), with a median of 131 comments (IQR: 35-318.5), 400 saves (IQR: 90.75-1,654.5), and 194.5 shares (IQR: 37.75-1,046.75). Content creators of the TikTok videos had a median follower count of 11,400 (IQR: 2,241-45,400). The median engagement index was 0.44 (IQR: 0.06-5.46). Content quality, as assessed by both human reviewers and AI using the GQS (Figure 1) and mDISCERN (Figure 2) instruments, was consistently rated at the lower end of the scoring range (median scores of 1-2 across all raters for both instruments).

**Figure 1:**
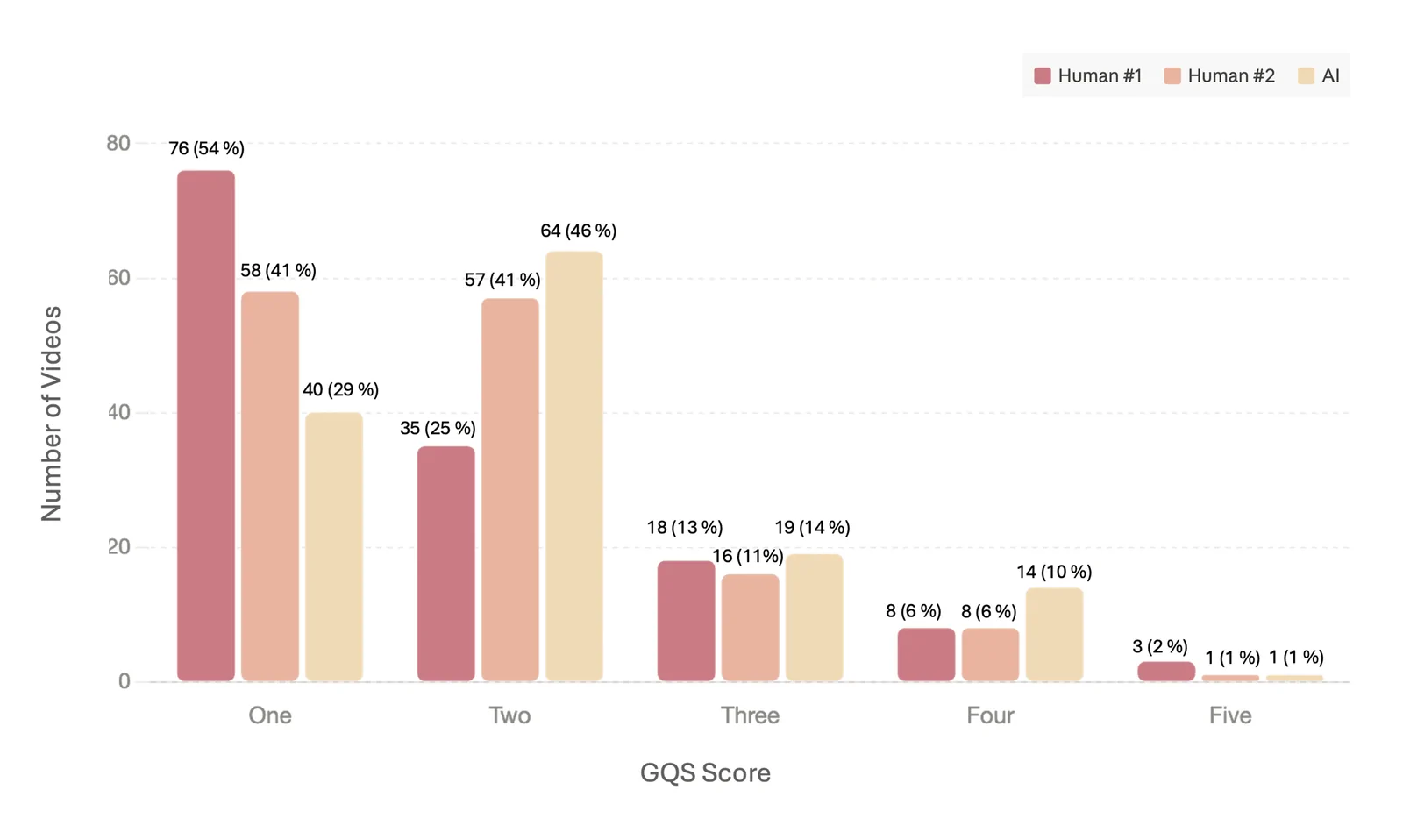
Distribution of GQS scores assigned by two human raters and an AI across 140 subjects. Human #1 assigned a score of "One" to the majority of items (54%), while the AI most frequently assigned a score of "Two" (46%), suggesting a more lenient quality assessment relative to the human raters. Human #2 showed an intermediate pattern, rating items equally as "One" or "Two" (41% each). GQS: Global Quality Scale.

**Figure 2:**
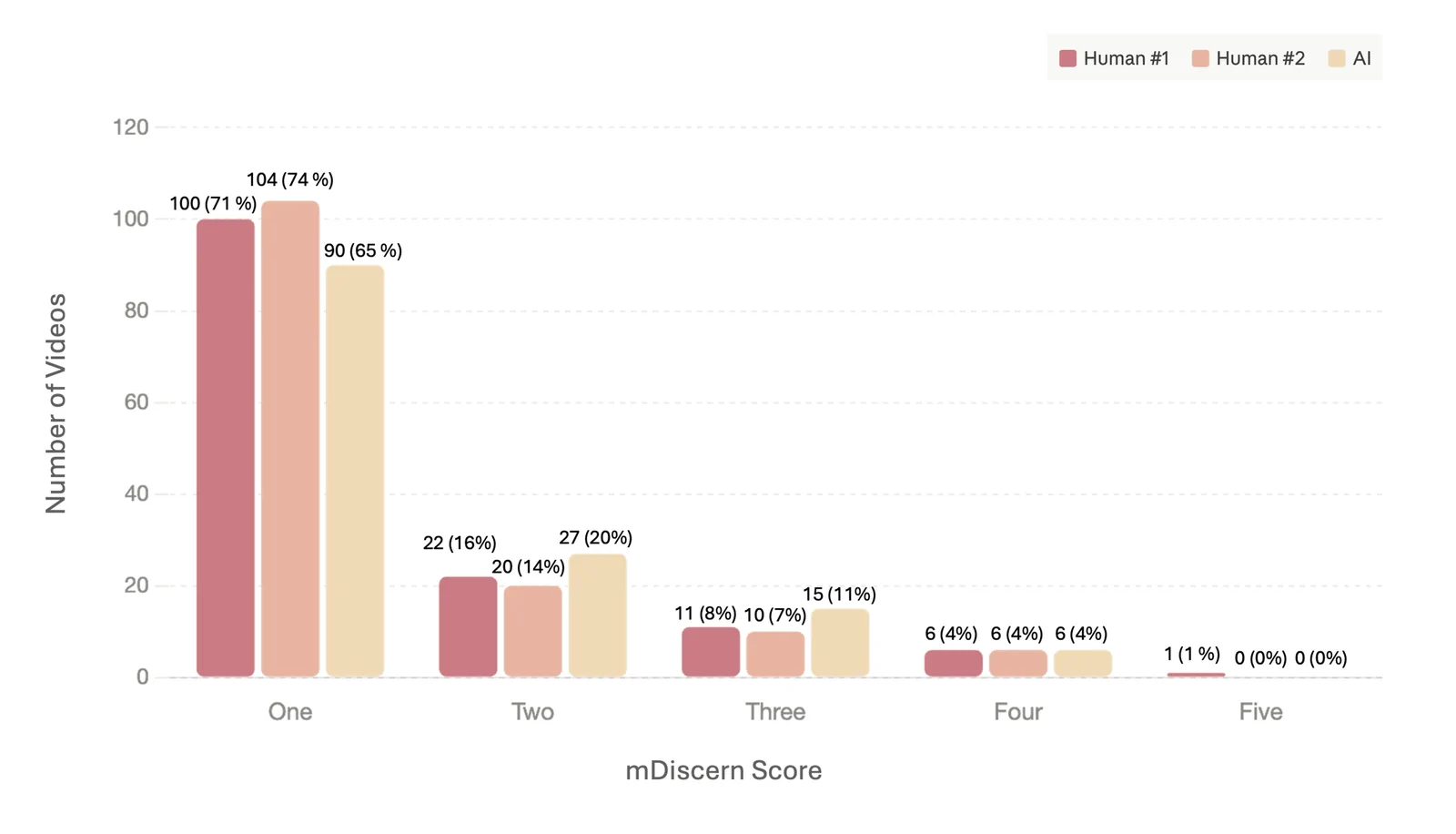
Distribution of mDISCERN scores assigned by two human raters and an AI across 140 subjects. The majority of items received a score of "One" across all three raters (Human #1: 71%, Human #2: 74%, AI: 65%), indicating generally low-quality health information. The AI tended to assign slightly higher scores compared to the human raters. mDISCERN: modified DISCERN.

Highly liked videos

When stratifying videos by engagement, those receiving 30,400 or more likes (upper quartile, 25% of videos) were compared with those receiving fewer than 30,400 likes (Table 2). Continuous variables are presented as median (IQR) unless otherwise specified. No statistically significant differences were observed between groups with respect to author type (Patient, 89% vs. 89%; Surgeon, 8% vs. 9%; p = 0.848; χ² = 0.81; df = 3), content type (Postoperative Experience, 72% vs. 83%; Education, 10% vs. 9%; p = 0.709; χ² = 2.15; df = 4), number of hashtags (3 (2-4) vs. 3 (2-5); p = 0.296; U = 1623.5), age of the video (250 (87-692.5) vs. 143 (57-533) days; p = 0.156; U = 1542), or follower count (13,500 (3,340-43,300) vs. 10,800 (1,896.25-48,100); p = 0.486; U = 1676). However, high-like videos were significantly shorter in duration (18 (13.5-40) vs. 34 (15-70) seconds; p = 0.016; U = 1335) and demonstrated markedly higher engagement across all metrics, including number of comments (879 (446.25-1,788.25) vs. 64 (21-170); p < 0.001; U = 135.5), saves (5,261 (1,622-11,550) vs. 198 (51-568); p < 0.001; U = 183), shares (4,745 (2,063.5-10,950) vs. 83 (31-286); p < 0.001; U = 74), shares/day index (43.45 (4.3-133.23) vs. 0.76 (0.12-2.13); p < 0.001; U = 363), and engagement index (10.9 (2.36-57.98) vs. 0.26 (0.05-0.89); p < 0.001; U = 652). Notably, high-like videos received lower content quality ratings from both human raters on the GQS (Human #1: 1 (1-1.5) vs. 2 (1-2), p = 0.010, U = 1349.5; Human #2: 1 (1-2) vs. 2 (1-2), p = 0.027, U = 1411), while AI-assessed GQS scores did not reach statistical significance (2 (1-2) vs. 2 (1.5-3); p = 0.055; U = 1449.5). The mDISCERN score assigned by Human #2 was also significantly lower in high-like videos (1 (1-1) vs. 1 (1-2); p = 0.013; U = 1423.5), whereas scores from Human #1 (1 (1-1) vs. 1 (1-2); p = 0.352; U = 1683.5) and AI (1 (1-1.5) vs. 1 (1-2); p = 0.148; U = 1552) did not differ significantly between groups. 

**Table 2:** Characteristics of videos that have been liked 30,400 times or more. ^†^Chi-square (χ^2^) test. ^§^Mann–Whitney U test. *The test statistic values appear in parentheses within the p-value column. GQS: Global Quality Scale; mDISCERN: modified DISCERN.

Variable	Number of likes per video		p-value*
	<30,400 Likes (75%)	>30,400 Likes (25%)	
Type of author^†^			0.848 (χ^2^ = 0.81)
Patient	93 (89 %)	31 (89 %)	
Surgeon	8 (8 %)	3 (9 %)	
Non-MD healthcare	2 (2 %)	1 (3 %)	
Activist	2 (2 %)	0 (0 %)	
Type of content^†^			0.709 (χ^2^ = 2.15)
Postoperative experience	76 (72 %)	29 (83 %)	
Education	11 (10 %)	3 (9 %)	
Preoperative experience	10 (10 %)	2 (6 %)	
Awareness/humor/support	5 (5 %)	1 (3 %)	
Procedure	3 (3 %)	0 (0 %)	
Duration of post (sec)^§^	34 (15-70)	18 (13.5-40)	0.016 (U = 1335)
Age of video (days)^§^	143 (57-533)	250 (87-692.5)	0.156 (U = 1542)
Likes^§^	1869 (455-5616)	93,500 (57,150-350,100)	<0.001 (U = 0)
Number of comments^§^	64 (21-170)	879 (446.25-1788.25)	<0.001 (U = 135.5)
Users saving post^§^	198 (51-568)	5261 (1622-11,550)	<0.001 (U = 183)
Hash-tags^§^	3 (2-5)	3 (2-4)	0.296 (U = 1623.5)
Shares^§^	83 (31-286)	4745 (2063.5-10,950)	<0.001 (U = 74)
Shares/day index^§^	0.76 (0.12-2.13)	43.45 (4.3-133.23)	<0.001 (U = 363)
Followers^§^	10,800 (1896.25-48,100)	13,500 (3340-43,300)	0.486 (U = 1676)
Engagement index^§^	0.26 (0.05-0.89)	10.9 (2.36-57.98)	<0.001 (U = 652)
GQS scores			
GQS Human #1^§^	2 (1-2)	1 (1-1.5)	0.01 (U = 1349.5)
GQS Human #2^§^	2 (1-2)	1 (1-2)	0.027 (U = 1411)
GQS AI^§^	2 (1.5-3)	2 (1-2)	0.055 (U = 1449.5)
mDISCERN scores			
mDISCERN Human #1^§^	1 (1-2)	1 (1-1)	0.352 (U = 1683.5)
mDISCERN Human #2^§^	1 (1-2)	1 (1-1)	0.013 (U = 1423.5)
mDISCERN AI^§^	1 (1-2)	1 (1-1.5)	0.148 (U = 1552)

Highly Shared Videos

A similar pattern was observed when videos were stratified by share count, comparing those with 1,050 or more shares (upper quartile) with those with fewer shares (Table 3). Continuous variables are presented as median (IQR) unless otherwise specified. Author type (Patient, 88% vs. 91%; Surgeon, 9% vs. 6%; p = 0.779; χ² = 1.09; df = 3), content type (Postoperative Experience, 71% vs. 86%; Education, 11% vs. 6%; p = 0.519; χ² = 3.24; df = 4), hashtag use (3 (2-4) vs. 3 (2-5); p = 0.386; U = 1659), video age (250 (70-692.5) vs. 143 (61-533) days; p = 0.277; U = 1611), and follower count (13,500 (2,769-40,950) vs. 10,800 (1,896.25-55,250); p = 0.684; U = 1735.5) did not differ significantly between groups. Highly shared videos were also significantly shorter (18 (14-40) vs. 34 (15-69) seconds; p = 0.027; U = 1379) and exhibited substantially greater overall engagement, including likes (93,500 (49,750-350,100) vs. 1,869 (455-5,616); p < 0.001; U = 40.5), comments (879 (392.25-1,788.25) vs. 64 (21-170); p < 0.001; U = 158.5), saves (5,261 (1,602.5-11,550) vs. 198 (51-568); p < 0.001; U = 190), shares (4,745 (2,063.5-10,950) vs. 83 (31-284); p < 0.001; U = 0), share/day index (43.45 (4.41-133.23) vs. 0.67 (0.12-2.13); p < 0.001; U = 268), and engagement index (12.9 (2.36-57.98) vs. 0.26 (0.05-0.89); p < 0.001; U = 582.5). Regarding content quality, high-share videos received significantly lower GQS ratings from both Human #1 (1 (1-1) vs. 2 (1-2); p = 0.003; U = 1267.5) and Human #2 (1 (1-2) vs. 2 (1-2); p = 0.016; U = 1374.5), while AI-generated GQS scores trended lower but did not reach statistical significance (2 (1-2) vs. 2 (1-3); p = 0.052; U = 1443.5). The mDISCERN score from Human #2 was significantly lower in high-share videos (1 (1-1) vs. 1 (1-2); p = 0.013; U = 1423.5), while scores from Human #1 (1 (1-1) vs. 1 (1-2); p = 0.303; U = 1667) and AI (1 (1-1) vs. 1 (1-2); p = 0.056; U = 1472.5) did not reach statistical significance. 

**Table 3:** Characteristics of videos that have been shared 1050 times or more. ^†^Chi-square (χ^2^) test. ^§^Mann–Whitney U test. *The test statistic values appear in parentheses within the p-value column. GQS: Global Quality Scale; AI: artificial intelligence; mDISCERN: modified DISCERN; χ²: chi-square statistic; U: Mann-Whitney U statistic.

Variables	Number of shares per video		p-value*
	<1050 Shares (75%)	>1050 Shares (25%)	
Type of author^†^			0.779 (χ^2^ = 1.09)
Patient	92 (88 %)	32 (91 %)	
Surgeon	9 (9 %)	2 (6 %)	
Non-MD healthcare	2 (2 %)	1 (3 %)	
Activist	2 (2 %)	0 (0 %)	
Type of content^†^			0.519 (χ^2^ = 3.24)
Postoperative experience	75 (71 %)	30 (86 %)	
Education	12 (11 %)	2 (6 %)	
Preoperative experience	10 (10 %)	2 (6 %)	
Awareness/humor/support	5 (5 %)	1 (3 %)	
Procedure	3 (3 %)	0 (0 %)	
Duration of post (sec)^§^	34 (15-69)	18 (14-40)	0.027 (U = 1379)
Age of video (days)^§^	143 (61-533)	250 (70-692.5)	0.277 (U = 1611)
Likes^§^	1869 (455-5616)	93500 (49,750-350,100)	<0.001 (U = 40.5)
Number of comments^§^	64 (21-170)	879 (392.25-1788.25)	<0.001 (U = 158.5)
Users saving post^§^	198 (51-568)	5261 (1602.5-11,550)	<0.001 (U = 190)
Hash-tags^§^	3 (2-5)	3 (2-4)	0.386 (U = 1659)
Shares^§^	83 (31-284)	4745 (2063.5-10,950)	<0.001 (U = 0)
Shares/day index^§^	0.67 (0.12-2.13)	43.45 (4.41-133.23)	<0.001 (U = 268)
Followers^§^	10,800 (1896.25-55,250)	13,500 (2769-40,950)	0.684 (U = 1735.5)
Engagement index^§^	0.26 (0.05-0.89)	12.9 (2.36-57.98)	<0.001 (U = 582.5)
GQS scores			
GQS Human #1^§^	2 (1-2)	1 (1-1)	0.003 (U = 1267.5)
GQS Human #2^§^	2 (1-2)	1 (1-2)	0.016 (U = 1374.5)
GQS AI^§^	2 (1-3)	2 (1-2)	0.052 (U = 1443.5)
mDISCERN scores			
mDISCERN Human #1^§^	1 (1-2)	1 (1-1)	0.303 (U = 1667)
mDISCERN Human #2^§^	1 (1-2)	1 (1-1)	0.013 (U = 1423.5)
mDISCERN AI^§^	1 (1-2)	1 (1-1)	0.056 (U = 1472.5)

Sentiment analysis

Sentiment analysis was performed on a total of 1,299 available comments across all included videos (Table 4). Both human reviewers identified the majority of comments as positive (Human #1: 52%; Human #2: 49%), with a minority classified as negative (Human #1: 9%; Human #2: 11%) and the remainder as neutral (Human #1: 39%; Human #2: 40%). AI-based sentiment classification using Claude yielded comparable results (positive: 58%; negative: 7%; neutral: 35%). In contrast, ChatGPT identified a substantially higher proportion of negative comments (23%) and a lower proportion of positive comments (46%), diverging from both human and Claude classifications. Inter-rater reliability between the two human reviewers demonstrated almost perfect agreement, with 1,222 observed agreements (94.07% of observations; κ = 0.898, SE = 0.011, 95% CI: 0.876-0.920). Agreement between the two AI systems was moderate, with 924 observed agreements (71.13% of observations; κ = 0.526, SE = 0.019, 95% CI: 0.489-0.562). 

**Table 4:** Sentiment classification comparing two human reviewers against AI across the complete comment dataset (1,299 comments).

Reviewer type	Sentiment	Reviewer 1	Reviewer 2	Kappa coefficient (95% CI)
Human reviewers		Human #1	Human #2	0.898 (0.876-0.920)
	Positive	675 (52%)	634 (49%)	
	Negative	118 (9%)	141 (11%)	
	Neutral	506 (39%)	524 (40%)	
Artificial intelligence		ChatGPT	Claude	0.526 (0.489-0.562)
	Positive	596 (46%)	753 (58%)	
	Negative	296 (23%)	93 (7%)	
	Neutral	407 (31%)	453 (35%)	

To assess AI accuracy against a consensus human standard, sentiment analysis was repeated on the 1,222 comments for which both human reviewers reached complete agreement (Table 5). The distribution of consensus human classifications remained consistent with the full dataset (positive: 52%; negative: 9%; neutral: 39%). Claude demonstrated close concordance with human consensus (positive: 58%; negative: 7%; neutral: 35%). ChatGPT, however, continued to overclassify negative sentiment (21%) and underclassify positive sentiment (48%) relative to the human consensus, suggesting a systematic difference in its sentiment classification approach compared with both human reviewers and Claude. When the dataset was narrowed to only those comments for which the two human reviewers were in full agreement on sentiment category, the concordance between ChatGPT and the human raters remained considerable, yielding a Cohen's kappa value of 0.649 (SE = 0.018; 95% CI: 0.614-0.684). Under the same conditions, Claude demonstrated even greater agreement with the human reviewers, achieving a Cohen's kappa coefficient of 0.749 (SE = 0.017; 95% CI: 0.715-0.783). 

**Table 5:** Sentiment analysis of the subset of comments on which both human reviewers were in unanimous agreement (1,222 comments). ^∆^Cohen’s kappa coefficient of 0.649 (SE = 0.018; 95%CI, 0.614–0.684). ^¶^Cohen’s kappa coefficient of 0.749 (SE = 0.017; 95%CI, 0.715–0.783).

Sentiment	Humans	ChatGPT^∆^	Claude^¶^
Positive	631 (52%)	586 (48%)	707 (58%)
Negative	111 (9%)	262 (21%)	91 (7%)
Neutral	480 (39%)	374 (31%)	424 (35%)

## Discussion

In this cross-sectional analysis of 140 TikTok videos related to breast reduction, content was overwhelmingly generated by patients and primarily focused on postoperative experiences. Overall engagement was substantial, with videos receiving thousands of likes, comments, saves, and shares despite generally poor informational quality as assessed by both human reviewers and artificial intelligence using validated evaluation instruments. Notably, videos with the highest levels of engagement, measured by likes and shares, were significantly shorter in duration and demonstrated markedly greater interaction across all engagement metrics. However, these highly engaging videos were also associated with lower content quality scores, suggesting that popularity may not correlate with the educational value or reliability of breast reduction information on TikTok. Sentiment analysis revealed that audience responses were predominantly positive, with strong agreement between human reviewers and AI-based classifiers. Collectively, these findings highlight TikTok's substantial reach as a source of breast reduction-related information while underscoring concerns regarding the quality of the content most likely to be disseminated and amplified by users.

Our findings differ substantially from those reported for breast augmentation content on TikTok [17]. While previous analyses demonstrated that breast augmentation videos generated substantially higher engagement, averaging 568,094 likes per video, our breast reduction cohort exhibited a median of 3,860.5 likes per video, suggesting lower overall audience reach and interaction [17]. Additionally, whereas plastic surgeons were the predominant creators of breast augmentation content (60%), breast reduction content was overwhelmingly patient-generated, with 89% of videos posted by patients and only 8% by surgeons [17]. Content themes also differed considerably; educational videos represented the most common category among breast augmentation videos (50%), whereas postoperative patient experiences accounted for three-quarters (75%) of breast reduction videos in our study. Despite these differences in creator type and content focus, both studies demonstrated similarly poor information quality [17]. Breast augmentation videos received a mean DISCERN score of 1.25, while breast reduction videos in our analysis consistently received median GQS and mDISCERN scores of 1-2 across both human and AI evaluators, indicating that low-quality information is pervasive across aesthetic breast surgery content on TikTok [17]. Notably, our study further demonstrated that videos with the highest levels of engagement were associated with significantly lower quality scores, suggesting that popularity on the platform may not reflect informational value and may instead favor brief, experience-driven content over evidence-based educational material [17].

An inverse relationship was observed in the present investigation between content quality, evaluated through both the GQS and the mDISCERN instrument, and measures of user engagement such as likes and shares. This counterintuitive phenomenon, whereby content of inferior quality is associated with greater reach and visibility across the platform, has been similarly identified in prior investigations examining surgical and aesthetic medicine content on social media. In a comparable analysis, Ravikumar and colleagues found that board-certified surgeons accounted for only a small minority of high-traffic plastic surgery content producers on TikTok and that their uploads, despite demonstrating superior accuracy and reliability, tended to generate comparatively less audience interaction than content produced by non-clinician creators [18]. These patterns carry substantial implications for public health, as they suggest that medically inaccurate or reductive content may exert a disproportionate influence on how patients form expectations and arrive at healthcare decisions [19].

Given that TikTok has been consistently characterized by elevated engagement alongside suboptimal informational quality, the results of the current study carry a dual significance [17,18]. On one hand, the limited presence of evidence-based, surgeon-authored content on the platform represents a largely untapped avenue through which plastic surgeons could meaningfully contribute to patient education using accurate, peer-reviewed material. On the other hand, the comparatively stronger engagement generated by lower-quality content highlights a systemic challenge: clinically rigorous and ethically responsible health information appears to be structurally disadvantaged within the algorithm-driven attention economy that governs content distribution on social media platforms, necessitating the development of more deliberate and effective outreach strategies by the medical community [20].

Regarding the sentiment analysis, the present study demonstrated that Claude achieved closer concordance with human consensus classifications than ChatGPT, a finding that contrasts with previous reports in which ChatGPT emerged as the superior model for sentiment and hesitancy analysis [21]. While both models achieved substantial agreement with human reviewers, Claude more faithfully replicated the distribution of human consensus classifications across all three sentiment categories. Consistent with prior literature, however, both models demonstrated meaningful limitations, particularly in the accurate classification of neutral sentiment, which remained the most challenging category across all raters [21]. Although 1,299 comments were analyzed, this remains a relatively small sample for sentiment analysis, and the comparative performance of the two models should be interpreted with caution until confirmed in larger datasets.

Taken together, these findings underscore that no current LLM can be considered uniformly superior for sentiment analysis in surgical research and that continued model refinement, with particular attention to nuanced linguistic constructs such as neutral affect, sarcasm, and indirect sentiment expression, remains essential before widespread clinical adoption can be recommended.

Limitations

First, the sample size was relatively small, only one search term ("Breast Reduction") was used to identify videos, and the search outcomes were influenced by the platform's proprietary algorithm, which may affect reproducibility. Although GQS and mDISCERN are validated instruments, they assess only selected dimensions of content quality and may not fully capture all aspects of educational value or informational accuracy. Sentiment analysis also has inherent constraints, as automated approaches may struggle to accurately identify sarcasm, irony, nuanced emotional expression, or context-dependent meaning. Additionally, the accuracy of transcripts generated by TikTok's AI-based tools may be affected by factors such as speech clarity, pronunciation, background noise, prosody, and articulation. Because both models were accessed through consumer chat interfaces, temperature and other AI generation parameters could not be set or viewed by the user, and default settings were used. This study examines associations rather than causal relationships.

Errors in transcription could subsequently influence downstream analyses, including sentiment classification and quality assessment. The cross-sectional design further limits the ability to evaluate temporal trends or changes in platform dynamics. Although the analysis was repeated across different accounts and networks and only identical classifications were retained, this approach improves consistency but does not establish reproducibility. Only publicly available content associated with breast reduction-related hashtags was analyzed; therefore, videos using colloquial terminology, alternative spellings, or nonstandard hashtags may have been overlooked. Moreover, TikTok's recommendation algorithms and the inclusion of only English-language videos may introduce selection bias by preferentially promoting content with greater engagement. Consequently, the engagement metrics observed in this study may reflect algorithmic amplification rather than the broader distribution of content available on the platform. Finally, this study evaluated TikTok only; findings may not generalize to other platforms such as Instagram, Facebook, or YouTube, whose users, content formats, and algorithms differ.

## Conclusions

Breast reduction content on TikTok is predominantly created by patients and primarily focuses on postoperative experiences, while overall informational quality remains low. Videos with the highest engagement were significantly shorter and received lower quality scores, suggesting that popularity is not associated with educational value. Public sentiment toward breast reduction was largely positive, and AI-based sentiment analysis demonstrated substantial agreement with human reviewers, particularly with Claude AI. Given TikTok's growing influence on perceptions of reduction mammaplasty, plastic surgeons and other healthcare professionals should take a more active role in creating and sharing accurate, evidence-based content to improve the quality and reliability of information available on social media.

## Appendices

Appendix 1

**Table 6:** Global Quality Scale criteria used to score videos containing information about reduction mammaplasty on TikTok.

Score	Global score description
1	Reflects very poor quality, characterized by disorganized presentation, substantial gaps in information, and minimal usefulness to patients.
2	Denotes low quality, with limited and poorly structured content where many critical topics are absent, offering little value to viewers.
3	Represents moderate quality, where some important information is adequately addressed but others are either incomplete or unclear, making it only partially helpful to patients
4	Indicates good quality, with generally well-organized and mostly comprehensive content, though a few relevant areas may still be lacking
5	Awarded to content that is exceptionally well-structured, complete, and highly informative, thereby serving as an excellent educational resource for patients

Appendix 2

**Table 7:** Modified DISCERN (mDISCERN) criteria used to score videos containing information about reduction mammaplasty on TikTok.

Item	Description: yes (1 score) / no (0 score)
1	Are aims clear and achieved?
2	Are reliable sources of information used?
3	Is the information provided balanced and unbiased?
4	Are additional sources of information listed for patient reference?
5	Any areas of uncertainty/controversy mentioned?

Appendix 3

Preconditioning of Comment Evaluators and AI

Directions: Please use the following guidelines to assess reactions from viewers commenting on a TikTok post concerning breast reduction, also known as reduction mammaplasty. Each comment should be categorized as expressing positive, neutral, or negative sentiment.

Positive sentiment: This category captures the emotional quality of a comment when it reflects happiness, optimism, or favorable impressions. Comments with positive sentiment tend to encourage the content creator to continue posting and reflect supportive feelings toward the material shared. In essence, it represents an overall tone of positivity conveyed through the language used.

Negative sentiment: This category captures text that reflects negative emotions, judgments, or viewpoints. It involves recognizing specific words, phrases, or overall messaging that carries an unfavorable or critical tone. Comments falling into this category imply that the viewer would prefer that the creator discontinue posting similar content.

Neutral sentiment: This classification applies when a comment lacks a distinctly positive or negative emotional charge. It reflects neither approval nor disapproval of the subject matter, instead presenting a balanced or objective viewpoint without a clear emotional leaning.
